# Recruiting migrant workers in Australia for Public Health surveys: how sampling strategy make a difference in estimates of workplace hazards

**DOI:** 10.1186/s13104-020-05320-x

**Published:** 2020-10-07

**Authors:** Alison Daly, Alison Reid

**Affiliations:** School of Public Health, Curtin University, GPO Box U1987, Perth, WA 6845 Australia

**Keywords:** Migrant workers, Cross-sectional surveys, Sampling, Methods

## Abstract

**Objectives:**

One third of the Australian work force are immigrants. Relatively little is known about working conditions for specific migrant groups. The objectives of this paper are to describe and compare the sampling strategies used to recruit migrant workers from specific migrant groups working in Australia into a cross-sectional study designed to produce population estimates of workplace hazards and self-reported health.

**Results:**

Two cross sectional telephone surveys were conducted with immigrants currently working in Australia. Survey 1 used quota sampling from lists provided by a sample broker. Survey 2 used a combination of probability and non-probability sampling, including random sampling from telephone lists. Data from the surveys were weighted and comparisons made with unweighted data. While weighting adjusted for most differences across the sample sources, the likelihood of exposure to workplace hazards depended on exposure types and sampling strategies. We concluded that by using a combination of sampling strategies it is possible to recruit immigrants from specific migrant groups and provide a balanced view of working conditions, although no one strategy was best for all types of measures. Access to a robust sample source for migrants would enable a better perspective to migrant populations’ working conditions.

## Introduction

Migrants who make up to 15% of workers in developed countries [1] have poorer working conditions than native-born populations [2–5]. They may also be in lower socioeconomic circumstances [6], have less access to health services including preventative services [7], poorer language proficiency [8], poorer health [9] and health literacy [10].

Research into the working conditions and health outcomes of foreign-born workers in Australia, who make up 35% of the Australian workforce [11], is limited. Data from 30 years ago showed that foreign-born workers, from non-English speaking backgrounds, had a higher risk of work-related fatality in the rural and mining industries than Australian-born, particularly in their first 5 years of residence in Australia [12]. Results from the few studies conducted in this area in Australia suggests that their job experience may differ from their Australian counterparts, both in terms of risks related to exposure to carcinogens [13–15] and exposure to workplace psychosocial stressors [16].

Recruitment of migrants into public health research is challenging. It has two major challenges. The first is the willingness of migrants to participate. While some research has found that migrants are as likely to participate in research as other groups [17], other research has reported a reluctance to talk about the research subject [18–20]. The second is the difficulty in finding a representative sample to enable population estimates that are reliable. The source of the sample has been shown to produce different estimates of exposure to workplace hazards depending on whether or not random sampling was used [21]. For minority population groups most researchers generally conclude that it not practicable to use purely probability sampling and advocate a variety of sampling strategies [22–24]. A systematic review found that even where it has been achieved, the non-response rate was relatively high with only four of seventeen reviewed studies having a non-response rate less than 20% [9]. The aim of this research note is to show how estimates of types of workplace hazards within migrant populations are affected by sample strategy types.

## Main text

### Materials and methods

Six of the larger minority ethnic groups in Australia, were selected for recruitment into two separate (S1 and S2) cross sectional computer assisted telephone interviews (CATI). CATI was chosen as it was to replicate previous work undertaken to examine workplace hazards [25]. Recruits for S1 were workers whose ancestry was Chinese, Arabic-speaking and Vietnamese. Recruits for S2 were workers born in New Zealand, India and the Philippines.

### Statistical analysis

Unweighted and weighted percentages were derived for socio economic and employment characteristics for both surveys. Iterative proportional fitting (IPF) [26] weighted the data using marginal proportions from the 2016 census for each migrant worker group [11]. Covariate adjusted logistic regression was used to develop models to compare sample characteristics for exposure to workplace hazards. Post estimation was conducted for goodness of fit [27] and final models used bootstrapped standard errors [28]. Analysis was done in Stata Version 14.2.

## Results

To achieve the 195 quota for each migrant group for S1, 19,300 numbers were called and the refusal rate (59.6%) was higher than the participation rate (40.4%) (Additional file 2). To achieve a sufficient sample size for each group for S2 over 300,000 numbers were called but the refusal rate for contacted households with eligible recruits was relatively low at 20% (Additional file 3). At least two-thirds of the sample for migrants born in New Zealand and India was recruited using random sampling from a known source. For migrants born in the Philippines this was 46.4% (Additional file 4). Numbers supplied by the sample broker for S1 were all landlines and for S2 were all mobile telephone numbers. Of the 59 recruited under ‘other sources’, 25 were mobile numbers and the remaining 34 were landlines (Table 1).

**Table 1:** Number of completed interviews by sample source and country of birth (S2), Australia 2017/2018

	New Zealand	India	Philippines
	n 566 (37.7%)	n 633 (38.8%)	n 431 (26.5%)
Original common surnames within suburbs	280 (49.5%)	319 (50.4%)	146 (33.9%)
High density suburb (common surnames names)	140 (24.7%)	97 (15.3%)	54 (12.5%)
Sample broker (unknown source)^a^	122 (21.6%)	196 (31.0%)	217 (50.4%)
Other sources, Australian survey^b^ and website to recruit	24 (4.2%)	21 (3.3%)	14 (3.3%)

^a^The sample provided by the sample broker contained only mobile numbers and no land lines. “Other sources” provided 34 landlines and 25 mobiles. The EWP samples were all landlines even though the sample frame provided contained some mobiles as potentially eligible contacts

^b^An earlier survey of Australian workers asked about recruits’ country of birth and those who were born in one of the migrant surveys target groups (and therefore ineligible for that study) were asked if they could be recalled if required. Of the 41 who consented, 13 were born in India, 23 born in New Zealand and 5 born in the Philippines

The unweighted estimates (Additional file 6) showed that the sample broker source (mobiles only) produced higher proportions of recruits that were younger, male and worked as machinery operators/labourers who had lived less time in Australia than recruits from any other sample source. The suburb density sampling sourced more metropolitan recruits. When the data were weighted, using age, gender, education and area of residence, almost all of the statistically significant percentage differences for gender and occupation were addressed (Additional file 7). However, even after weighting, there was still a greater percentage of younger recruits from the sample broker source (mobile only).

Additional file 5 shows the comparison between S1 and S2. Participants recruited via landline numbers are more likely to be aged 56–65 years and in managerial or professional occupations. Participants recruited via mobile numbers had lived in Australia for fewer years than participants recruited by landlines. Participants recruited by a sample broker using landline numbers were in the occupations grouped under technician/community services/clerical and sales. The biggest differences between the 2016 Census and the samples were India-born workers aged 26–35 years (sample 21.6% vs census 47.3%) and New Zealand females (sample 58% vs census 36.7%). With these exceptions, the weighted percentages were more representative of each migrant worker population, independent of the sample source and telephone type.

Table 2 shows the odd ratios for exposure to workplace hazards. There were no statistically significant differences in the likelihood of reporting three or more psychosocial adversities by sample type, telephone type or sample source. However, there was a decreased likelihood of exposure to workplace carcinogens of almost one half for respondents probability sampled compared with respondents who were called using any other sampling strategy. When telephone types were compared, respondents called on mobiles had an increased likelihood of exposure to at least one of ten carcinogens.

**Table 2:** Odd ratios for psychosocial (S1 and S2) and carcinogen exposure (S2 only) by sampling sources

Sample type	Perceived exposure to 3 or more psychosocial adversities				Exposed to at least 1 of 10 workplace carcinogens^a^			
	OR (95% CI)	*p*	aOR^b^ (95% CI)	*p*	OR (95% CI)	*p*	aOR^b^ (95% CI)	*p*
Non probability (Recall)	1.00		1.00		1.00		1.00	
Probability (EWP based random sampling)	1.31 (0.99, 1.73)	0.06	1.3 (0.92, 1.85)	0.14	0.59 (0.47, 0.74)	< 0.001	0.67 (0.5, 0.89)	0.006
Study phone								
Landline S1 (Sample broker)	1.00		1.00					
Landline S2 (EWP based random sampling)	1.29 (0.94, 1.76)	0.12	1.5 (0.8, 2.82)	0.20	1.00		1.00	
Mobile S2 (Sample broker)	1.04 (0.69, 1.58)	0.84	1.26 (0.62, 2.54)	0.52	1.77 (1.4, 2.22)	< 0.001	1.54 (1.16, 2.05)	0.003
Sample source								
Original surname sample (landline)	1.00		1.00		1.00		1.00	
Suburb based sample (landline)	1.22 (0.86, 1.75)	0.27	1.41 (0.95, 2.09)	0.09	0.78 (0.6, 1.01)	0.06	1.41 (0.95, 2.09)	0.09
Sample broker (mobile)^c^	0.81 (0.6, 1.11)	0.20	0.86 (0.57, 1.28)	0.45	1.63 (1.29, 2.07)	< 0.001	1.45 (1.09, 1.93)	0.01
Recall (both)	0.72 (0.25, 2.08)	0.54	0.74 (0.25, 2.17)	0.59	1.14 (0.71, 1.83)	0.58	1.11 (0.59, 2.08)	0.74

^a^Exposure to carcinogens reported only from Survey 2

^b^Models were adjusted for age, sex, area of residence, education, country of birth, weekly hours worked, whether employed full or part-time and occupation

^c^The same sample broker was used to provide sample for both S1 and S2 but only provided mobile numbers for S2

## Discussion

There were big differences in response rates for S1 and S2. This is puzzling as the same company conducted the interviews for both surveys and the same sample broker was used for both surveys. The only major difference between the two surveys was the ethnic ancestry of the migrant groups studied. It may be that different cultures have different attitudes towards participating in surveys. All the details about the sampling strategies and outcomes from these are found in our Additional file 1: Table S1, Additional file 2: Figure S1, Additional file 3: Figure S2, Additional file 4: Table S2 and Additional file 5: Table S3. The description of the sample with unweighted and weighted estimates are found in Additional file 6: Table S4 and Additional file 7: Table S5.

For adverse workplace psychosocial hazards such as perceived job insecurity or high job demand, it does not appear to matter whether or not probability sampling is used, whether or not mobiles are used instead of landlines or where the sample came from. However, when estimates of the likelihood of exposure to workplace carcinogens is the subject of the investigation, then these differences do matter. Probability sampling showed statistically decreased likelihood of exposure whereas being called on a mobile or having the sample provided by a sample broker showed statistically increased likelihood of exposure, even when adjusted for covariates including age, sex and occupation.

Sampling strategies used to investigate minority groups in populations in cross sectional studies usually involved a variety of non-probability recruitment strategies including purposive and convenience sampling [18, 29, 30]. Less common were cross sectional surveys of working sub population groups using probability sampling. One of these, the Spanish National Health Survey used a probability sampled cross sectional survey to assess the Spanish working population. While 8591 workers responded, only 711 (0.08%) were immigrant workers [31]. Another study designed to compare a household based sampling method with the census for a particular region of the US, took 14 months to recruit immigrant participants and required extraction of additional immigrant households but was successful in identifying a representative sample of the immigrant population of the area [32]. In our study, the attempt to obtain participants through probability random sampling resulted in an extremely high number of telephone calls being made. This proved to be time consuming and costly. The numbers provided by the sample broker resulted in more efficient recruitment but from unknown source(s). Across all strategies, once eligible participants were found, they were easily recruited with very few refusals and little unused sample, in contrast to S1.

The major problem with non-probability sampling, such as purposive and convenience sampling, is that while these can provide a great deal of information about a particular sub group, with some exceptions [33, 34], prevalence estimates from these sampling methods are not easy to generalise to a population [17, 33]. For S1, we had no information about the sample provided by the sample broker other than the fact that all the telephone numbers were landlines. For S2, we knew that just under half of our respondents came from the EWP (n 745, 45.7%) but that the sample broker source was more successful in sourcing young male migrant workers, who are often less likely to participate in research [35, 36]. The persistent smaller mean number of years in Australia from mobile phone recruits while obviously linked to age, may also reflect a preference for migrant workers to use mobile telephones but this would need to be investigated. Furthermore, adjustment for occupation in our models did not fully explain the disparities observed in mobile phone recruits who were more likely to have worked as machine operators and labourers and to have been exposed to carcinogens in the workplace. We did test for interactions between occupation, age and phone type but there were no statistically significant associations. There is likely to be residual confounding when we adjust for occupation and because mobile 'pay as you go' phones are cheaper than landlines, we may be inadvertently measuring an indicator of lower socio demographic status.

More innovative methods, such as propensity matching scoring [37], need to be explored but the best source of sample would be access to sample frames such as those available for public health emergencies in Australia [38].

## Conclusion

This study demonstrated that robust population based estimates for different migrant worker groups are possible when a variety of sampling methods are used and proper weighting procedures applied to the data. The study also showed that there may be groups that are not adequately represented when sub populations estimates are made, particularly those who use only mobile phones. There is a strong public health case to be made for access to an adequate sample frame and the development of appropriate methods to reach migrant workers.

## Limitations

The use of the EWP as a source of sampling frame was a limitation due to the lack of mobile telephone numbers that are listed. The strength of using the EWP as a sample frame was the ability to undertake probability sampling and therefore provide more robust estimates at a population level.

Samples provided by sample brokers come from unknown sources and make the ability to both weight and generalise a challenge. A strength of the sample supplied by the sample broker was the ability to capture mobile phone users who proved to be younger and not working in the manager/professional occupations making the total sample more representative of the working population in Australia.

## Supplementary information

** Additional file 1.** Sampling strategies and outcomes in recruiting migrant workers into two cross-sectional surveys in a population setting.

**Additional file 2: Figure S1.** Response flow chart for Study One (S1).

**Additional file 3: Figure S2.** Response flow chart for Study Two (S2).

**Additional file 4.** Number of completed interviews by sample source and country of birth (S2), Australia 2017/18.

**Additional file 5.** S2 unweighted and weighted percentages of socio demographic and occupation groups of survey recruits by telephone type, Australia 2017/18.

**Additional file 6.** Unweighted estimates with 95% CI for socio demographic and employment variables by sample source, Australia 2017/18.

**Additional file 7.** Weighted estimates with 95% CI for socio demographic and employment variables by sample source, Australia 2017/18.

## Data Availability

The datasets used and/or analysed during the current study are available from the corresponding author on reasonable request.

## Abbreviations

EWP: Electronic White Pages
S1: Survey one
S2: Survey two
