# Supplementary material for: Recruiting migrant workers in Australia for Public Health surveys: how sampling strategy make a difference in estimates of workplace hazards
Source: BMC Res Notes. 2020 Oct 7;13:473. doi: 10.1186/s13104-020-05320-x (PMC7542909; doi:10.1186/s13104-020-05320-x)
Supplement: Supplementary file 1 — Additional file 1. Sampling strategies and outcomes in recruiting migrant workers into two cross-sectional surveys in a population setting. [file 13104_2020_5320_MOESM1_ESM.docx]

**Additional file 1 Sampling strategies and outcomes in recruiting migrant workers into two cross-sectional surveys in a population setting**

|  | Strategy | Outcome |
| --- | --- | --- |
| Study One (S1 2015) | Both the Electronic White pages (EWP) and a sample broker were used to provide a sample frame of telephone numbers from three major Australian cities selected by surnames that were common to the target minority* groups. Random samples were then selected and called until quotas were reached. | From initial sample of 36956 numbers provided, 17656 numbers were unused; Total numbers called contacted=9898  Aged 18 plus, currently working & in one of the target groups=1443  Interviewed=585  Refused=863 |
| Study Two (Strategy 1 2017/18) | Census data was used to identify possible suburbs with migrant** residents in the EWP. Surnames common to the target minority groups were extracted and used as the sample frame. Random samples for each migrant group were selected and every number was called. | **From this strategy**, 745 of the 1630 were recruited (45.7%) |
| Study Two (Strategy 2) | The previously identified suburbs with migrant residents from the EWP were filtered so that only suburbs with the highest density of migrants in the target groups were extracted and used as the sample frame. Random samples for each migrant group were selected and every number was called. | **From this strategy**, 291 of the 1630 were recruited (17.9%) |
| Study Two (Strategy 3) | A sample broker provided samples of telephone numbers for the targeted migrant groups with commonly used– mobile numbers only were supplied. Random samples for each migrant group were selected and all but 313 numbers were called. | **From this strategy,** 535 were recruited (32.8%) |
| Study Two (Strategy 4) | A webpage was created; information was sent out to community groups and churches; influential members of the target minority groups were contacted; and snowballing was used once a contact had been established. 25 were contacted by mobile | **From this strategy,** 59 of 1630 were recruited (3.6%) |

*the first study selected on the basis of identification with an ethnic background rather than country of birth

**the second study selected on the basis of country of birth
