# Supplementary material for: Recruiting migrant workers in Australia for Public Health surveys: how sampling strategy make a difference in estimates of workplace hazards
Source: BMC Res Notes. 2020 Oct 7;13:473. doi: 10.1186/s13104-020-05320-x (PMC7542909; doi:10.1186/s13104-020-05320-x)
Supplement: Supplementary file 2 — Additional file 2: Figure S1. Response flow chart for Study One (S1). [file 13104_2020_5320_MOESM2_ESM.docx]

Total unused sample

17656 (47.8%)

Total sample provided

36956

Unable to determine eligibility (no answer)

13,401 (69.4%)

Numbers called 19300 (52.2%)

**Raw Response rate =30.6%**

Contacted numbers

5,899 (30.6%)

Ineligible

4348 (73.7%)

Eligible

1,551 (26.3%)

**Eligible Response rate 37.8%**

Appointment made but not kept as study ended =103 (6.6%)

Not aged 18-65

3,060 (70.4%)

Not working

764 (17.6%)

**Additional file 2, Figure S1. Response flow chart for Study One (S1)**

Raw response rate = completed interviews/total numbers used; Eligible response rate=completed interviews/total numbers contacted; Participation response rate =completed interviews/total eligible contacted

Unable to determine language

89 (2.0%)

Called for interview

1448 (93.4%)

Interviewed

585 (40.4%)

Refused

863 (59.6%)

Ethnic quota full

397 (9.1%)

**Participation rate**

**40.4%**

Incapacitated

38 (0.9%)
