# Supplementary material for: Recruiting migrant workers in Australia for Public Health surveys: how sampling strategy make a difference in estimates of workplace hazards
Source: BMC Res Notes. 2020 Oct 7;13:473. doi: 10.1186/s13104-020-05320-x (PMC7542909; doi:10.1186/s13104-020-05320-x)
Supplement: Supplementary file 3 — Additional file 3: Figure S2. Response flow chart for Study Two (S2). [file 13104_2020_5320_MOESM3_ESM.docx]

Unable to determine eligibility (no answer)

225748 (72.7%)

Total sample provided

310636

Contacted numbers

84,888 (28.3%)

**Raw Response rate**

**27.3%**

Ineligible

85,254 (97.2%)

Eligible

2,364 (2.8%)

Eligible from sample broker but no more required for that migrant group

313 (13.2%)

**Eligible Response rate**

**69%**

Not aged 18-65

8,484 (70.4%)

Not working

20,048 (17.6%)

**Participation rate**

**79.5%**

Interviewed

1630 (79.5%)

Refused

420 (20.5 %)

Called for interview

2,051 (86.8%)

**Additional file 3, Figure S2. Response flow chart for Study Two (S2)**

Raw response rate = completed interviews/total numbers used; Eligible response rate=completed interviews/total numbers contacted; Participation response rate =completed interviews/total eligible contacted

Incapacitated

64 (0.9%)

Not in migrant target group 53,594

Unable to determine language

334 (2.0%)
