# Supplementary material for: Recruiting migrant workers in Australia for Public Health surveys: how sampling strategy make a difference in estimates of workplace hazards
Source: BMC Res Notes. 2020 Oct 7;13:473. doi: 10.1186/s13104-020-05320-x (PMC7542909; doi:10.1186/s13104-020-05320-x)
Supplement: Supplementary file 4 — Additional file 4. Number of completed interviews by sample source and country of birth (S2), Australia 2017/18. [file 13104_2020_5320_MOESM4_ESM.docx]

**Additional file 4 Number of completed interviews by sample source and country of birth (S2), Australia 2017/18**

|  | New Zealand | India | Philippines |
| --- | --- | --- | --- |
|  | **n 566 (37.7%)** | **n 633 (38.8%)** | **n 431 (26.5%)** |
| Original common surnames within suburbs | 280 (49.5%) | 319 (50.4%) | 146 (33.9%) |
| High density suburb (common surnames names) | 140 (24.7%) | 97 (15.3%) | 54 (12.5%) |
| Sample broker (unknown source)^a^ | 122 (21.6%) | 196 (31.0%) | 217 (50.4%) |
| Other sources, Australian survey^b^ & website to recruit | 24 (4.2%) | 21 (3.3%) | 14 (3.3%) |

a The sample provided by the sample broker contained only mobile numbers and no land lines. “Other sources” provided 34 landlines and 25 mobiles. The EWP samples were all landlines even though the sample frame provided contained some mobiles as potentially eligible contacts.

b An earlier survey of Australian workers asked about recruits’ country of birth and those who were born in one of the migrant surveys target groups (and therefore ineligible for that study) were asked if they could be recalled if required. Of the 41 who consented, 13 were born in India, 23 born in New Zealand and 5 born in the Philippines.
