# Supplementary material for: Recruiting migrant workers in Australia for Public Health surveys: how sampling strategy make a difference in estimates of workplace hazards
Source: BMC Res Notes. 2020 Oct 7;13:473. doi: 10.1186/s13104-020-05320-x (PMC7542909; doi:10.1186/s13104-020-05320-x)
Supplement: Supplementary file 5 — Additional file 5. S2 unweighted and weighted percentages of socio demographic and occupation groups of survey recruits by telephone type, Australia 2017/18. [file 13104_2020_5320_MOESM5_ESM.docx]

**Additional file 5 S2 unweighted and weighted percentages of socio demographic and occupation groups of survey recruits by telephone type, Australia 2017/18**

|  | **S1a**  **(sample broker landlines 99%)** | **S2**  **(sample broker- mobile only)** | **S2**  **(EWP landline only)** | **S1a**  **(sample broker landlines 99%)** | **S2**  **(sample broker- mobile only)** | **S2**  **(EWP landline only** |
| --- | --- | --- | --- | --- | --- | --- |
|  | **Unweighted** | | | **Weighted** | | |
| **Age group** |  |  |  |  |  |  |
| 18-25 | 12.1 (9.5,15.2) | 7.7 (5.8,10.3) | **3.7 (2.7,5.0)^b^** | 11.4 [7.7,16.4] | 10.8 [7.7,14.9] | 10.0 [7.1,13.9] |
| 26-35 | **19.5 (16.3,23.1)** | **28.5 (24.9,32.4)** | 10.5 (8.8,12.5) | 25.6 [19.7,32.6] | ***43.6 [38.4,48.9]^c^*** | 22.9 [19.1,27.2] |
| 36-45 | 22.0 (18.6,25.8) | **34.4 (30.6,38.5)** | 26.0 (23.5,28.7) | 26.7 [21.3,32.8] | 26.1 [22.3,30.2] | 27.3 [24.1,30.8] |
| 46-55 | 28.4 (24.7,32.5) | **20.5 (17.4,24.1)** | 33.1 (30.3,35.9) | 25.7 [20.9,31.1] | ***13.9 [11.2,17.1]*** | 22.9 [19.9,26.1] |
| 56 -65^a^ | 18.1 (15.0,21.7) | **8.8 (6.7,11.5)** | **26.8 (24.2,29.5)** | 10.7 [8.0,14.2] | 5.7 [4.0,8.0] | ***16.9 [14.5,19.6]*** |
| **Gender** |  |  |  |  |  |  |
| Male | 49.2 (45.2,53.3) | **60.7 (56.6,64.7)** | 48.8 (45.8,51.8) | 56 [49.6,62.2] | 64.8 [59.9,69.5] | 57.7 [53.7,61.7] |
| Female | 50.8 (46.7,54.8) | **39.3 (35.3,43.4)** | 51.2 (48.2,54.2) | 44 [37.8,50.4] | ***35.2 [30.5,40.1]*** | 42.3 [38.3,46.3] |
| **Occupation** |  |  |  |  |  |  |
| Manager/Professional | 33.4 (29.7,37.3) | 31.3 (27.5,35.2) | **43.4 (40.4,46.4)** | 22.0 [17.6,27.2] | 30.2 [25.8,34.9] | ***36.9 [33.2,40.7]*** |
| Technician/community services/clerical/sales | 46.3 (42.3,50.4) | 49.3 (45.2,53.4) | 44.2 (41.3,47.2) | ***55.5 [49.1,61.7]*** | 46.6 [41.6,51.7] | 43.9 [39.8,48.1] |
| Machinery operators /Labourer | 20.3 (17.2,23.8) | 19.5 (16.4,23) | **12.4 (10.6,14.5)** | 22.4 [17.6,28.1] | 23.2 [18.8,28.3] | 19.2 [15.7,23.2] |
| **Mean years in Australia** | 21.9 (20.9,23.0) | **12.0 (11.2,12.8)** | 20.0 (19.2,20.7) | 20.5 (19.1,21.9) | ***10.9 (10.0,11.7*)** | 17.4 (16.6,18.3) |

a There were five people who were over the age of 65 and these were coded back into age 56-65 years

b The bolded figures indicate significant differences for the unweighted prevalence estimates.

c The bolded and italicised figures indicate significant differences for the weighted prevalence estimates
