# Supplementary material for: Recruiting migrant workers in Australia for Public Health surveys: how sampling strategy make a difference in estimates of workplace hazards
Source: BMC Res Notes. 2020 Oct 7;13:473. doi: 10.1186/s13104-020-05320-x (PMC7542909; doi:10.1186/s13104-020-05320-x)
Supplement: Supplementary file 6 — Additional file 6. Unweighted estimates with 95% CI for socio demographic and employment variables by sample source, Australia 2017/18. [file 13104_2020_5320_MOESM6_ESM.docx]

**Additional file 6 Unweighted estimates with 95% CI for socio demographic and employment variables by sample source, Australia 2017/18**

|  | **Random suburb based (n=745)** | **High density suburb^a^ (n=291)** | | **Sample broker (n=535)** | | **Various methods (n=59)** | |
| --- | --- | --- | --- | --- | --- | --- | --- |
| **Age group** |  |  |  | |  | |  |
| 18-25 | 4.2 [2.9,5.9] | 2.8 [1.4,5.5] | **8.1 [6.1,10.8]** | | 0 | |  |
| 26-35 | 11.0 [9,13.5] | 9.4 [6.5,13.3] | **28.3 [24.6,32.3]** | | 18.6 [10.6,30.7] | |  |
| 36-45 | 27.5 [24.4,30.8] | 21.5 [17.1,26.7] | 34.7 [30.8,38.9] | | 30.5 [20.1,43.4] | |  |
| 46-55 | 31.8 [28.5,35.2] | 35.8 [30.4,41.5] | 20.6 [17.3,24.2] | | 30.5 [20.1,43.4] | |  |
| 56 -65^b^ | 25.6 [22.6,28.8] | 30.6 [25.5,36.1] | **8.3 [6.2,11.0**] | | 20.3 [11.9,32.6] | |  |
| **Gender** |  |  |  | |  | |  |
| Male | 50.7 [47.1,54.3] | 45.4 [39.7,51.1] | **60.9 [56.7,65**] | | 44.1 [31.9,57] | |  |
| Female | 49.3 [45.7,52.9] | 54.6 [48.9,60.3] | 39.1 [35,43.3] | | 55.9 [43,68.1] | |  |
| **Area of residence** |  |  |  | |  | |  |
| Metro | 80.5 [77.5,83.2] | 89.7 [85.6,92.7] | 75.3 [71.5,78.8] | | 64.4 [51.4,75.6] | |  |
| Rest of State | 19.5 [16.8,22.5] | **10.3 [7.3,14.4]** | 24.7 [21.2,28.5] | | 35.6 [24.4,48.6] | |  |
| **Education** |  |  |  | |  | |  |
| Up to year 12 or equivalent | 12.5 [10.3,15.1] | 15.5 [11.7,20.1] | 14.0 [11.3,17.3] | | 15.3 [8.1,26.9] | |  |
| Diploma/Trade | 28.8 [25.7,32.2] | 27.5 [22.7,32.9] | 29.2 [25.5,33.2] | | 23.7 [14.5,36.3] | |  |
| Tertiary | 58.7 [55.1,62.2] | 57.0 [51.3,62.6] | 56.7 [52.5,60.9] | | 61.0 [48.0,72.6] | |  |
| **Employment type** |  |  |  | |  | |  |
| Works for others | 87.8 [85.2,89.9] | 83.8 [79.2,87.7] | 86.7 [83.6,89.4] | | 88.1 [77,94.3] | |  |
| Self-employed | 12.2 [10.1,14.8] | 16.2 [12.3,20.8] | 13.3 [10.6,16.4] | | 11.9 [5.7,23.0] | |  |
| **Contract type** |  |  |  | |  | |  |
| Casual | 15.7 [13.2,18.5] | 12.1 [8.7,16.5] | 18.4 [15.3,22.0] | | 7.4 [2.8,18.3] | |  |
| Fixed term part-time | 1.8 [1.0,3.1] | 1.8 [0.8,4.3] | 1.9 [1.0,3.6] | | 0 | |  |
| Fixed term full-time | 3.7 [2.6,5.4] | 4.8 [2.8,8.0] | 6.6 [4.8,9.1] | | 3.7 [0.9,13.8] | |  |
| Permanent | 78.8 [75.7,81.7] | 81.3 [76.2,85.5] | 73 [69.0,76.7] | | 88.9 [77.2,95] | |  |
| **Occupation** |  |  |  | |  | |  |
| Manager/Professional | 42.8 [39.3,46.4] | 44.0 [38.4,49.8] | **31.2 [27.4,35.3]** | | 42.4 [30.4,55.3] | |  |
| Technician/community services/clerical/sales | 43.2 [39.7,46.8] | 47.8 [42.1,53.5] | 49.5 [45.3,53.8] | | 39.0 [27.4,52.0] | |  |
| Machinery operators/Labourer | 14.0 [11.6,16.6] | 8.2 [5.6,12.0] | **19.3 [16.1,22.8]** | | 18.6 [10.6,30.7] | |  |
| **Mean years in Australia** | 19.4[18.6,20.3] | 21.1[19.8,22.4] | 11.8[11,12.7] | | 19.6[16.8,22.3] | |  |

a High density suburbs were those with a high density of surnames common to each migrant target group and were mainly in the metropolitan areas of the states.

b There were five people who were over the age of 65 and these were coded back into age 56-65 years
