# Supplementary material for: Recruiting migrant workers in Australia for Public Health surveys: how sampling strategy make a difference in estimates of workplace hazards
Source: BMC Res Notes. 2020 Oct 7;13:473. doi: 10.1186/s13104-020-05320-x (PMC7542909; doi:10.1186/s13104-020-05320-x)
Supplement: Supplementary file 7 — Additional file 7. Weighted estimates with 95% CI for socio demographic and employment variables by sample source, Australia 2017/18. [file 13104_2020_5320_MOESM7_ESM.docx]

**Additional file 7 Weighted estimates with 95% CI for socio demographic and employment variables by sample source, Australia 2017/18**

|  | **Random suburb based (n=745)** | **High density suburb^a^ (n=291)** | | **Sample broker (n=535)** | | **Various methods (n=59)** | | ***p*** | |
| --- | --- | --- | --- | --- | --- | --- | --- | --- | --- |
| **Age group** |  |  |  | |  | |  | |  |
| 18-25 | 11.7 [8.1,16.7] | 6.5 [2.9,13.9] | 11.2 [8.0,15.4] | | 0 | |  | |  |
| 26-35 | 23.1 [18.7,28.2] | 21.2[14.9,29.3] | 43.3 [38.0,48.8] | | 39.1[20.6,61.4] | |  | |  |
| 36-45 | 28.4 [24.5,32.6] | 22.3 [17.1,28.5] | 26.2 [22.3,30.4] | | 33.9 [19.2,52.5] | |  | |  |
| 46-55 | 22.1 [18.5,26.1] | 25.8 [20.5,32.0] | 13.9[11.1,17.2] | | 16.0 [8.5,28.2] | |  | |  |
| 56 -65^b^ | 14.7 [12.3,17.4] | 24.1 [18.3,31.1] | 5.4 [3.7,7.9] | | 11.0[5.4,21.2] | | <0.0001 | |  |
| **Gender** |  |  |  | |  | |  | |  |
| Male | 60.1 [55.4,64.6] | 53.5 [45.9,60.8] | 64.9 [59.8,69.6] | | 50.5 [31.6,69.2] | |  | |  |
| Female | 39.9 [35.4,44.6] | 46.5 [39.2,54.1] | 35.1 [30.4,40.2] | | 49.5 [30.8,68.4] | | 0.076 | |  |
| **Area of residence** |  |  |  | |  | |  | |  |
| Metro | 79.4 [74.6,83.4] | 92.4 [87.6,95.4] | 79.7 [75.3,83.4] | | 65.9 [48.3,80.0] | |  | |  |
| Rest of State | 20.6 [16.6,25.4] | 7.6 [4.6,12.4] | 20.3 [16.6,24.7] | | 34.1 [20.0,51.7] | | 0.0005 | |  |
| **Education** |  |  |  | |  | |  | |  |
| Up to year 12 | 27.3 [22.1,33.1] | 26.5 [19.3,35.2] | 25.3 [20.1,31.3] | | 35.8 [17.2,59.8] | |  | |  |
| Diploma/Trade | 22.0 [18.7,25.6] | 23.6 [18.1,30.1] | 21.9 [18.4,25.9] | | 17.7 [8.7,32.6] | |  | |  |
| Tertiary | 50.7 [45.8,55.7] | 49.9 [42.4,57.4] | 52.8 [47.4,58.0] | | 46.6 [29.0,65.1] | | 0.899 | |  |
| **Employment type** |  |  |  | |  | |  | |  |
| Works for others | 88.6 [84.8,91.5] | 86.1 [81.1,90.0] | 87.3 [82.8,90.8] | | 88.1 [72.7,95.3] | |  | |  |
| Self-employed | 11.4 [8.5,15.2] | 13.9 [10.0,18.9] | 12.7 [9.2,17.2] | | 11.9 [4.7,27.3] | | 0.874 | |  |
| **Contract type** |  |  |  | |  | |  | |  |
| Casual | 19.5 [15.6,24.1] | 14.3 [9.2,21.5] | 19.9 [16.0,24.5] | | 7.9 [2.8,20.6] | |  | |  |
| Fixed term part-time | 2.0 [1.0,3.8] | 1.8 [0.5,6.0] | 1.8 [0.8,3.8] | | 0 | |  | |  |
| Fixed term full-time | 3.4 [2.0,5.7] | 3.3 [1.7,6.2] | 6 [4.1,8.6] | | 4.9 [1.2,18.3] | |  | |  |
| Permanent | 75.1 [70.3,79.3] | 80.6 [73.3,86.3] | 72.3 [67.5,76.7] | | 87.2 [72.5,94.6] | | 0.340 | |  |
| **Occupation** |  |  |  | |  | |  | |  |
| Manager/Professional | 36.1 [31.8,40.6] | 39.6 [32.6,47.1] | 30.1 [25.6,34.9] | | 33.3 [19.7,50.4] | |  | |  |
| Technician/community services/clerical/sales | 43.1 [38.2,48.1] | 46.8 [39.4,54.3] | 46.8 [41.6,52.1] | | 40.8 [22.5,62.1] | |  | |  |
| Machinery operators/Labourer | 20.8 [16.7,25.7] | 13.6 [8.1,22.0] | 23.1 [18.6,28.4] | | 25.9 [12.9,45.1] | | 0.242 | |  |
| **Mean years in Australia** | 16.4 [15.4,17.4] | 20.1 [18.3,21.9] | 10.8 [9.9,11.6] | | 16.7 [13.9,19.5] | |  | |  |

a High density suburbs were those with a high density of surnames common to each migrant target group and were mainly in the metropolitan areas of the states.

b There were five people who were over the age of 65 and these were coded back into age 56-65 years
